# Applications of analysis of dynamic adaptations in parameter trajectories

**DOI:** 10.1098/rsfs.2012.0084

**Published:** 2013-04-06

**Authors:** Natal A. W. van Riel, Christian A. Tiemann, Joep Vanlier, Peter A. J. Hilbers

**Affiliations:** 1 Department of Biomedical Engineering, Eindhoven University of Technology, Den Dolech 2, Eindhoven, 5612 AZ, The Netherlands; 2 Institute for Complex Molecular Systems, Eindhoven University of Technology, Den Dolech 2, Eindhoven, 5612 AZ, The Netherlands; 3 Netherlands Consortium for Systems Biology, University of Amsterdam, Science Park 904, Amsterdam, 1098 XH, The Netherlands

**Keywords:** systems biology, parameter estimation, uncertainty analysis, multi-scale, metabolic networks, progressive disease

## Abstract

Metabolic profiling in combination with pathway-based analyses and computational modelling are becoming increasingly important in clinical and preclinical research. Modelling multi-factorial, progressive diseases requires the integration of molecular data at the metabolome, proteome and transcriptome levels. Also the dynamic interaction of organs and tissues needs to be considered. The processes involved cover time scales that are several orders of magnitude different. We report applications of a computational approach to bridge the scales and different levels of biological detail. Analysis of dynamic adaptations in parameter trajectories (ADAPTs) aims to investigate phenotype transitions during disease development and after a therapeutic intervention. ADAPT is based on a time-dependent evolution of model parameters to describe the dynamics of metabolic adaptations. The progression of metabolic adaptations is predicted by identifying necessary dynamic changes in the model parameters to describe the transition between experimental data obtained during different stages. To get a better understanding of the concept, the ADAPT approach is illustrated in a theoretical study. Its application in research on progressive changes in lipoprotein metabolism is also discussed.

## 1. Introduction

In preclinical and clinical research, different stages of a disease can be phenotyped by collecting information of the genome, proteome, microbiome, etc. Likewise, the effect of therapeutic interventions can be analysed in longitudinal studies. The data at each stage provide a *snapshot* of the phenotype [1]. To integrate and interpret this multivariate data, systems biology approaches, such as pathway-based analyses and computational modelling, are becoming increasingly important [2,3]. Despite the progress in bioinformatics and computational systems biology, novel computational approaches are necessary to exploit the full potential of the information contained in the data.

Differential equation models can capture causal relationships in biomolecular reaction networks and describe system dynamics [4,5]. Many established numerical methods are available to simulate and analyse such models. We hypothesized that computational modelling using differential equations provides a suitable basis for the development of an approach to link phenotype snapshots as a function of time, hereby providing an integrated understanding of disease progression. In our approach, several challenges are faced. First, understanding and modelling disease progression such as in cancer and type 2 diabetes needs to consider the multiple factors involved. On the one hand, molecular data at the metabolome, proteome and transcriptome levels should be integrated (the field of molecular systems biology). On the other hand, the dynamic interactions of organs and tissues should be considered (physiology). Secondly, dynamic computational models in biology are typically constructed to simulate processes at a single time scale, usually capturing short-term dynamics ranging from seconds to hours [4,6,7]. However, diseases typically develop and progress over a period of years, and also therapeutic interventions take considerable time to become effective. The time scales of the molecular mechanisms governing cell behaviour versus the gradual and adaptive (patho)physiological changes induced by a progressive disease differ many orders of magnitude. The presence of these largely different time scales imposes serious challenges for the well-established modelling approaches. Thirdly, a major challenge in computational systems biology is to obtain values for parameters such that the model adequately represents the *in vivo* system. Quantitative biological data needed as input for model development are still relatively sparse, despite many innovations in measurement technologies. Because of the uncertainties associated with the data and the complexity of the system at hand, in general, multiple acceptable parameter values are obtained. Here acceptable means that the parameter values yield a model that is in agreement with the experimental data. It is important to analyse how uncertainties in experimental data propagate through parameter values and model predictions. The last two topics have triggered important new developments in parameter estimation and computational statistics [8,9].

We have developed a computational approach which contributes to a solution for the challenges describe earlier. The approach, called Analysis of Dynamic Adaptations in Parameter Trajectories (ADAPTs), aims to model and analyse phenotype transitions during disease progression and after a therapeutic intervention [10]. With ADAPT, models are developed that can describe long-term adaptations in a biological system involving the interactions between metabolome, proteome and transcriptome, despite the lack of mechanistic details regarding the regulation of metabolic processes by changes in gene and protein expression. In addition to the long-term adaptations, the same model can also simulate short-term dynamics (e.g. on a time scale of seconds) induced by variations in metabolite concentrations and fluxes. The finite accuracy of experimental data and the typical experimental restriction that only a subset of the model variables can be measured are sources for uncertainty. Errors and simplifications in the model are an important second type of uncertainty that is analysed in ADAPT. The latter, referred to as ‘undermodelling’ [11], represents primarily the missing information regarding the regulation by the transcriptome and proteome.

The ADAPT approach is outlined in §2. Subsequently, the mathematical framework and computational methods are described. To get a better understanding of ADAPT, it was applied in a theoretical study. After the results for the case study are presented, the application in a study of progressive changes in lipoprotein metabolism is discussed. An abnormal amount of lipids (e.g. cholesterol and/or fat) in blood and changes in lipoprotein composition (dyslipidemia) is an important hallmark of metabolic syndrome and recognized as a risk factor for type 2 diabetes [12].

## 2. Approach

**Figure 1.**
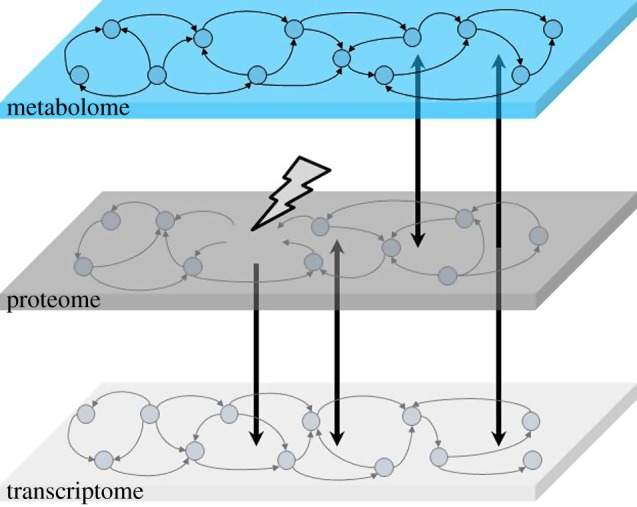
The ADAPT approach is founded at the metabolic level. The topology of metabolic networks is mostly known. The metabolic interaction network is shown as a directed graph. Also the kinetics of many of the interactions (enzymatic reactions) in metabolic pathways have been studied. The interaction networks of genes and proteins are less well known. However, an intervention at one level will result in changes in the other levels as well (the vertical arrows). ADAPT does not model changes in gene expression and protein activity mechanistically, but these interactions are incorporated by introducing time-varying parameters in the description of the metabolic network. (Online version in colour.)

The foundation of the ADAPT approach is a mathematical model of the metabolic networks in cells, between cells in different tissues and in the blood plasma connecting the tissues (figure 1). ADAPT integrates the large body of work on the biochemistry of metabolic pathways with metabolomic data. The metabolic network is described with a high level of structural complexity. The network topology is available for different organisms in several pathway/genome databases, such as listed in Pathguide (http://www.pathguide.org). The model includes biochemical kinetics and can describe metabolite concentrations and fluxes. The model is parametrized with kinetic information from literature and databases, where metabolite profiling data are used to estimate unknown or uncertain model parameters. Transitions between metabolic phenotypes during disease progression also involve changes in the transcriptome and proteome. Likewise, interventions at one level (e.g. adding a protein kinase inhibitor drug) will result in changes in the other levels as well (figure 1). However, the interaction networks of genes and proteins are less well known, and kinetic information is generally lacking. At this moment, it is not yet feasible to include a mechanistic description of this level of regulation in the model. ADAPT uses an innovative approach to overcome this problem.

By collecting data from different stages of disease progression, different realizations of the model are obtained. If it is assumed that the structure of the metabolic network is invariant, then the different clinical phenotype snapshots translate into different sets of parameter values, each characterizing a specific disease stage. In case of a progressive disease, we can also assume that the phenotype snapshots are temporarily related, and therefore also the corresponding models should have such a relation. Given the invariant structure of the metabolic network, this relation is captured by the difference in the values of the kinetic parameters of the different models. We therefore postulated that the adaptations in the metabolic network, as a result of a progressing disease or intervention, can be described with parameters that vary as function of time. The time-varying parameters can be represented as trajectories in the mathematical space spanned by the parameter vectors. Using the constraints imposed by the network structure (mass balances and conservation of mass), kinetic rate equations and the metabolomics snapshot data per phenotype we can infer trajectories of the model parameters which link the different phenotypes into a model of disease progression. We hypothesize that the parameter trajectories have a biological interpretation and reflect changes in the proteome and transcriptome that modulate metabolic concentrations and fluxes. The computational methods to implement ADAPT are described in detail in §3.

## 3. Material and methods

**Figure 2.**
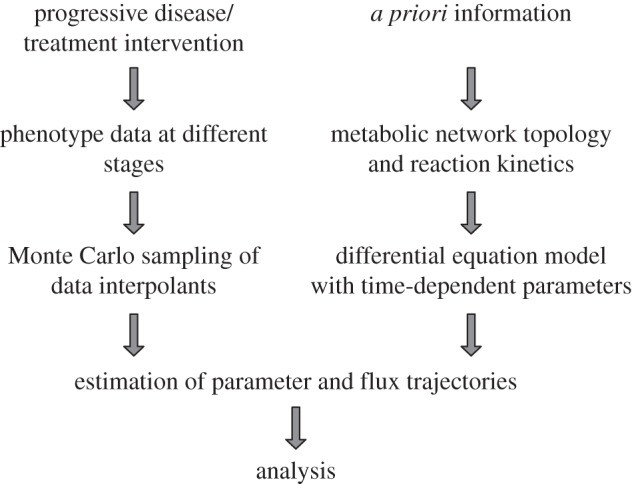
Outline of the Analysis of Dynamic Adaptations in Parameter Trajectories (ADAPT) approach.

An overview of the ADAPT approach is shown in figure 2. Details about the mathematics and numerical methods have been described in Tiemann *et al*. [10,13] and are repeated here in short for clarity and consistency.

### 3.1. Differential equation models

We start from the common approach to describe the dynamics of a biological system by a following set of (non)linear ordinary differential equations (ODEs):3.1
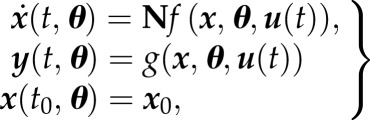


where 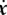 is a vector of first derivatives of molecular species ***x*** which are given by the topology of the network (matrix **N**) and a set of functions *f*. The initial concentrations of ***x*** are given by ***x***_0_. The vector ***y*** represents the model outputs, which are described by a set of functions *g*. Both functions *f* and *g* depend on kinetic parameters ***θ*** and time-dependent inputs ***u***(*t*).

### 3.2. Time-dependent descriptions of model parameters

Dynamic models according to equation (3.1) have been well established in modelling metabolic networks, although equation (3.1), in principle, can describe any biomolecular reaction network. The topology of metabolic networks is much better known than for gene and protein networks. For many metabolic pathways also kinetic information is available, which is generally lacking at the transcriptome and proteome levels. In ADAPT, the modulating effects on the metabolic pathways via interactions with the proteome and transcriptome levels are captured with more crude approximations by time-dependent descriptions of the parameters. The mathematical formulation in equation (3.1) is extended by introducing a time-dependency of the parameters ***θ***:3.2
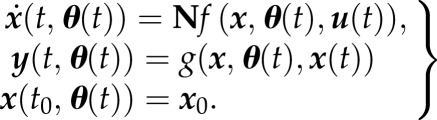


Note that it is not known *a priori* how the model parameters change during progression of the disease. Consequently, it is not possible to perform a dynamic simulation of the entire time span in one go. Latter issue is addressed by simulating the system in a step-wise manner. Each step a simulation is performed from current time *t* to time *t*+*δt*, using the values of ***x***(*t*) as initial conditions. Simultaneously, parameters ***θ***(*t*+*δt*) are estimated each step, using ***θ***(*t*) as initial set, by minimizing the difference between experimental data and corresponding model outputs ***y***(*t*+*δt*). It is assumed that the induced adaptations proceed progressively in time. Highly fluctuating parameter trajectories are considered to be unphysiological. To prevent the occurrence of such behaviour, a regularization term, given by the sum of squared derivatives of the normalized parameter values at current time *t*, is included in the parameter estimation procedure. An optimized parameter set, denoted by ***θ̂***(*t*), is defined as3.3
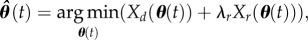
where *λ_r_* is a constant determining the strength of the regularization term. A minimal value for *λ_r_* is chosen to bias the data fitting as little as possible [10]. The objective functions *X_d_* and *X_r_* are given by3.4
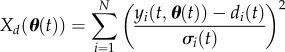
and3.5
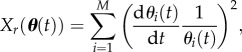
where *X_d_* is the sum of squared errors (SSEs), *N* the number of measurements, *M* the number of parameters, ***d*** the experimental data and ***σ*** the corresponding standard deviations.

### 3.3. Experimental data and bootstrap sampling of interpolants

To enable the estimation of dynamic trajectories of metabolic parameters and fluxes according to equation (3.3), continuous dynamic descriptions of the experimental data are required as input for the computational method. For this purpose, cubic smoothing splines were calculated that describe the dynamic trend of the experimental data ***d*** [14]. To account for experimental and biological uncertainties, a bootstrapping approach was used. In bootstrapping, replicates of the observed data are sampled, and the estimation is repeated for each of these samples [15–20]. Bootstrap samples of the data were obtained from a parametric error model distribution, assuming Gaussian distributions with means and standard deviations of the data. For each generated sample, a cubic smoothing spline was calculated, resulting in a distribution of data interpolants.

### 3.4. Numerical algorithms

The mathematical model and parameter estimation routines were implemented in Matlab (2011b, The MathWorks, Natick, MA, USA). Software has been developed to reduce the computation time for the simulation of ODE models in Matlab (CVode Wrapper, http://bmi.bmt.tue.nl/sysbio/software/pua.html, [8]). The CVode Wrapper package for Matlab includes a parser to convert a Matlab m-file containing the ODEs to a C-file, and compile the C-source file and the numerical integrators from the SUNDIALS CVode package (2.6.0, Lawrence Livermore National Laboratory, Livermore, CA, USA [21]) into a MEX file that can be run in Matlab. The ODEs were solved with an absolute and relative tolerance of 10^−6^. The Matlab nonlinear least-squares solver LSQNONLIN, which uses an interior reflective Newton method [22], was used to estimate model parameters. Both the termination tolerances for the objective function and the parameter estimates were set to 10^−6^. The Matlab function CSAPS was used to calculate the cubic smoothing splines.

## 4. Case study

### 4.1. Rationale

To get a better understanding of the approach, to test the computational algorithms and to identify possibilities for further improvements, ADAPT was applied in a theoretical study using a ‘toy model’. A model was used as the system under study. In three cases, different parameter inference problems have been studied. Starting with a ‘classical’ problem of estimating a parameter in a kinetic model and finally applying ADAPT. With ADAPT, a model was developed that is able to describe long-term dynamic changes in response to an intervention as well as the short-term dynamics at any time during the progressive adaptation. This includes stages in between the experimentally observed phenotypes. This theoretical study allowed comparison of the ADAPT results with results of the true system.

A small system composed of a metabolic network regulated by transcriptional feedback was studied. To incorporate adaptations in the system that operate at time scales much larger than the dynamics of metabolic reactions (seconds) and gene expression (hours), the transcription and translation rates were changed in a step-wise manner (see table 5 in appendix B) to create five different phenotypes and five corresponding datasets. However, in the analyses, this change in the system was treated as an unknown external intervention, progressively changing the transcriptome level. In all cases, the same experimental data were used.

#### 4.1.1. Case 1

First, it was analysed whether changes in the transcriptional feedback could be identified at different stages of the intervention. It was assumed that only metabolite concentrations could be measured, and a classical parameter estimation approach was used to investigate whether parameters of the gene circuit could be (re)estimated. The parameters of the metabolic network were kept fixed (known).

#### 4.1.2. Case 2

Secondly, we considered the situation in which we lacked any information about the transcriptional feedback mechanism, but only knew which pathway in the metabolic network was affected by the intervention. The model in this case was significantly simpler than the true system (undermodelling). To incorporate the effect of the intervention, we hypothesized that at the metabolic level this effect can be reproduced by changing (kinetic) parameters in the metabolic network. In this case, these metabolic parameters lump enzyme kinetics and possible regulation through changes in transcription, translation and post-translational modification. For each of the five datasets, separate parameter values were inferred.

#### 4.1.3. Case 3

In case 2, the different phenotype snapshots were translated into different distributions of parameter values. However, the parameter values (and hence also the resulting models) were disjoint from each other. In addition to the prior knowledge that the structure of the metabolic network is the same for all stages after the intervention, we could also assume that the phenotype snapshots are temporarily related, and therefore also the corresponding models should have such a relation. However, this information cannot be used in the modelling approach applied in case 2. In case 3, ADAPT was applied to link the different phenotypes in a consistent way and obtain a model able to describe all metabolic data at all stages using time-varying parameters.

### 4.2. System and models

#### 4.2.1. System

**Figure 3.**
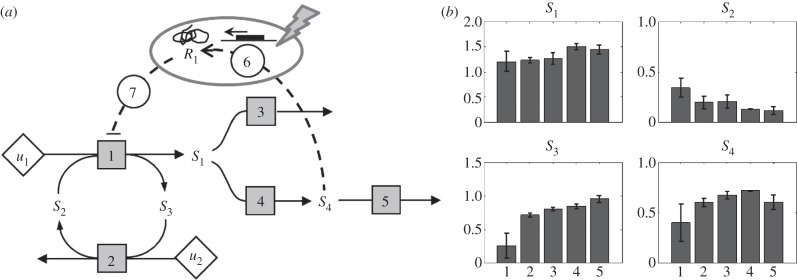
As case study a metabolic system with metabolite controlled, negative transcriptional feedback was used. (*a*) A schematic representation of the system. The metabolic network is regulated through a negative feedback gene circuit. An external intervention is applied affecting the transcriptome and/or proteome level of the system. The square boxes and solid lines represent biochemical reactions, *S*_1_–*S*_4_ are intracellular metabolites and *u*_1_ and *u*_2_ are input fluxes. Reactions 6 and 7 marked with circles represent transcription and translation, and inhibition, respectively. *R*_1_ is the mRNA of the regulator. (*b*) Metabolic phenotyping at five different stages (1, … ,5) of the system that progressively adapts to the intervention (the long-term dynamics). The intervention directly affects the transcriptome–proteome levels, which results in an adaption of the metabolic network, as is clear from the differences in the steady-state metabolite concentrations. The error bars indicate the standard deviation of the data.

The metabolic network consists of a conserved moiety cycle and a branch (figure 3). *S*_1_–*S*_4_ are intracellular metabolites and *u*_1_ and *u*_2_ are input fluxes. The metabolic network is regulated through a negative feedback gene circuit. An increase in downstream metabolite *S*_4_ results in the inhibition of an upstream metabolic pathway. The activity of enzymes in metabolic pathways can be inhibited by, for example, covalent modification (phosphorylation). This mechanism resembles catabolite repression in micro-organisms [23,24]. An external intervention has been applied affecting the transcriptome and/or proteome level of the system. Typical perturbations can be a pharmacological intervention, or a gene knockdown with siRNA. Reactions 6 and 7 in figure 3 represent transcription and translation, and enzyme inhibition, respectively. *R*_1_ is the mRNA of the regulator.

The system is described according to4.1
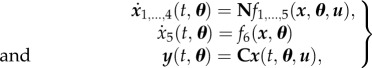
where the state variables are ***x*** = [*S*_1_,*S*_2_,*S*_3_,*S*_4_,*R*_1_]^T^, input fluxes ***u*** = [*u*_1_,*u*_2_,]^T^ and **N** is the stoichiometric matrix of the metabolic network4.2
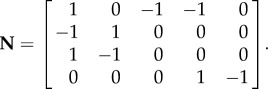


The rate equations *f* describe the kinetics4.3
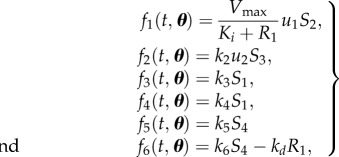
with parameter vector ***θ*** = [*V*_max_, *K*_i_,*k*_2_,*k*_3_,*k*_4_,*k*_5_,*k*_6_,*k_d_*]. Mass action kinetics was used except for *f*_1_, which describes enzyme inhibition, with *K_i_* the binding affinity of the inhibitor.

The observation matrix **C**,4.4
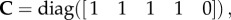
indicates that the metabolites are observable outputs of the system, but the mRNA of the regulator *R*_1_ is not. Values for parameters, input fluxes and initial conditions ***x***_0_ are provided in appendix A.

#### 4.2.2. Data

The system was studied at five different stages during its progressive adaptation to the external intervention. The stages have been indicated as 1, … ,5, in which 1 represents the basal, reference phenotype. It was assumed that during each examination the system in equation (4.1) was in steady-state (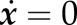). The metabolic profile was taken as the steady-state values of the outputs ***y***_ss_ (the phenotype snapshot). To generate the ‘mock’ experimental data noise was added4.5
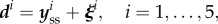
where *i* refers to the observations of the different stages and ***ξ****^i^* a noise sequence with a zero mean Gaussian distribution and a standard deviation ***σ****^i^*. ***σ****^i^* was chosen between 0 and 20 per cent of the output for the reference phenotype (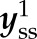), randomly generated from a uniform distribution. The data used here are shown in figure 3*b*.

To translate this data model to a clinical situation, one could think of a patient of whom the reference condition is determined at day 1. Next, a therapeutic intervention is applied and the effects are observed by measuring the patient's metabolic profile once per day for four subsequent days. All observations are done under fasting conditions, i.e. at a physiological steady-state.

#### 4.2.3. Case 1

The model was described according to equations (4.1)–(4.3); however, parameter *k*_6_, the rate constant representing transcription and translation, was affected by the intervention and unknown. A least-squares method (minimizing a weighted SSEs between model and data according to equation (3.3) with *λ_r_* = 0) was used to estimate a value for *k*_6_ for each metabolic phenotype, resulting in four estimates for 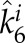 (*i* = 2, … ,5). The value for *k*_6_ in the reference condition was known and used as the initial value (guess) to start the numerical optimization algorithm to estimate the value for the other datasets. The only uncertainty that needed to be considered was due to the noise in the experimental data.

#### 4.2.4. Case 2

The transcriptional regulation of reaction 1 in the scheme of figure 3*a* was considered unknown, but it was assumed to be known that the intervention had changed the flux through reaction 1. The analysed model consisted of the metabolic network only (no information about the transcription/translation process was incorporated):4.6
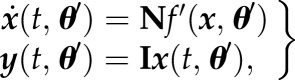
where *f*′ = *f*_1, … ,5_ in equation (4.3), but with *f*_1_ according to equation (4.7). **I** is the identity matrix, indicating that all state variables (metabolite concentrations) are outputs of the model.4.7
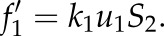


The parameter vector is ***θ***′ = [*k*_1_,*k*_2_,*k*_3_,*k*_4_,*k*_5_]. The introduced parameter *k*_1_ is a lumped parameter used to capture and approximate the combined processes of *f*_1_ and *f*_6_ in the true system (equation (4.3)). This parameter was estimated for each phenotype independently. In contrast to case 1, here two sources of uncertainty were present in the inference problem. There was the noise in the data, but the model was also a simplified description of reality (undermodelling). A Monte Carlo type of approach was used to estimate *k*_1_, with bootstrapping from a parametric error model for the data, resulting in a distribution of estimated values for each phenotype. The data distributions were sampled and the least-squares algorithm was run 1000 times.

#### 4.2.5. Case 3

Here the power of the ADAPT approach has been exploited to integrate the data of the different phenotypes in a single model. The metabolic model is described with time-dependent parameters4.8
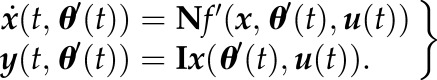
Again it was assumed to be known that the intervention had affected reaction 1, and it was analysed whether the different phenotypes could be linked by introducing a time-dependent *k*_1_(*t*). For *k*_1_(*t*), a parameter trajectory was inferred from the joint experimental data. The other parameters were assumed to be fixed, hence ***θ***′(*t*) = [*k*_1_(*t*),*k*_2_,*k*_3_,*k*_4_,*k*_5_].

An ensemble of parameter trajectories was inferred by repeating the ADAPT algorithm 1000 times. For each run, a new spline interpolant was fitted through a bootstrap sample of the data to get a continuous function for the data. Each time, a different sample was drawn from the distributions of the experimental data. Because of the complexity of the optimization problem, there was a fair chance that some runs of the parameter trajectory analysis got stuck in some local optimum in parameter space for which the corresponding model cannot describe the data accurately. This is reflected by a large SSE value (*X_d_* in equation (3.3)). If a run did not yield an SSE below a certain threshold, this result was discarded. To initiate, the trajectory an initial value was randomly chosen from a log-uniform distribution between 0.1 and 10, and the parameter was optimized to describe the data of stage 1 (the reference phenotype). The settings used for the ADAPT algorithms can be found in table 1.

**Table 1.** Settings for the ADAPT algorithm.

parameter	description	value
*λ_r_*	Lagrange multiplier weighting the regularization of parameter changes versus datafit	0.1
*δt*	time interval for the step-wise integration and data interpolants	0.1
lr	lower bound for the range of which initial values for the parameters are sampled	0.1
ur	upper bound for the range of which initial values for the parameters are sampled	10
nr	number of repeats/samples of the parameter trajectories	1000
sseThres	threshold for SSE to accept or reject parameter values	1000

## 5. Results

### 5.1. Model including transcriptional feedback

**Figure 4.**
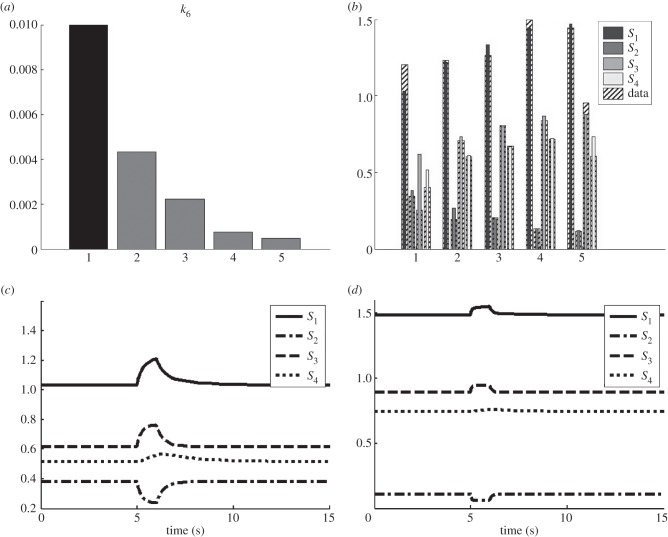
Case 1, estimate transcription and translation rate constant *k*_6_ in the model for the different stages after the intervention. (*a*) The parameter at stage 1 (before the intervention) was known (black), the parameter values at stages 2, … ,5 have been estimated (grey). (*b*) Comparison of the four metabolites in the model (*S*_1_, … ,*S*_4_, grey scale bars) with the data (dashed bars in the back). (*c*) The short-term dynamics (seconds time scale) of the original metabolic system (stage 1) in response to a pulse in input flux *u*_1_ (at *t* = 5 s). (*d*) The short-term dynamics of the model in stage 5 in response to a pulse in the input flux.

As a first case, it was investigated whether a parameter describing transcription and translation could be accurately estimated from steady-state metabolite concentration data. For reference phenotype 1, the rate constant 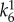 for transcription and translation was known (equal to 0.01, table 2 in appendix A). Values for *k*_6_ in the four other phenotypes were estimated from the metabolic data for each stage by a least-squares approach. The results in figure 4 identified a progressive decrease in *k*_6_. The variance in the estimates 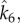 approximated from the inverse of the Fisher Information Matrix [25], was small (results not shown). Moreover, the estimates were also accurate, being close to the true values of *k*_6_ with which the data were generated (listed in table 5 in appendix A). The five resulting models, only different in the value for *k*_6_, could each describe the corresponding data accurately (figure 4*b*). The difference between model and data and the (minor) uncertainty in parameter values was due to noise (variance) in the experimental data.

Although the models have been parametrized by fitting the steady state to the phenotype data, the resulting models are dynamic. The models can predict responses to variations in concentrations or fluxes, such as a pulse in one of the input fluxes. A small pulse (short duration and low amplitude) results in temporally different metabolite concentrations and fluxes. The feedback via gene regulation is not activated and the original steady-state is rapidly recovered (within seconds). Figure 4*c* shows the short-term dynamics of the original metabolic system (stage 1) in response to a pulse in *u*_1_ (at *t* = 5 s). The system recovered steady-state within 10 s and the transcriptional feedback was not activated (*R*_1_ remained constant, not shown). The different operational setpoints of the system at the five stages (different steady-states) were also reflected in differences in the short-term dynamic responses to a pulse in *u*_1_. For comparison, the response of the model at stage 5 is shown (figure 4*d*). Note that the kinetic parameters of the metabolic network were the same for all five stages.

### 5.2. Independent parametrization for each phenotype

**Figure 5.**
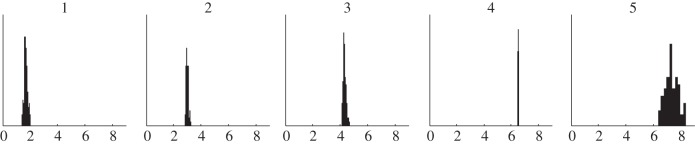
Case 2, estimation of the effect of the intervention on the lumped metabolic parameter *k*_1_ at five different stages. Distributions of the parameter have been obtained by a Monte Carlo approach.

For case 2, the progressive adaptation in metabolism was captured in the lumped parameter *k*_1_. This parameter was estimated for the five different phenotypes using a Monte Carlo approach. The resulting distributions for *k*_1_ are shown in figure 5. The identified decrease in *k*_6_ for case 1 means the inhibition of the first reaction in the metabolic network is elevated resulting in a larger flux. In case 2, it is shown that the phenomenological parameter *k*_1_ can reproduce the same behaviour in the metabolic network. In addition to the gradual increase in the average value of *k*_1_, there is a difference in the precision (variance) of the estimated values. Especially in stage 4, the variance is much smaller than for the other data. This can partly be explained from the low variance in the data of stage 4 (figure 3).

**Figure 6.**
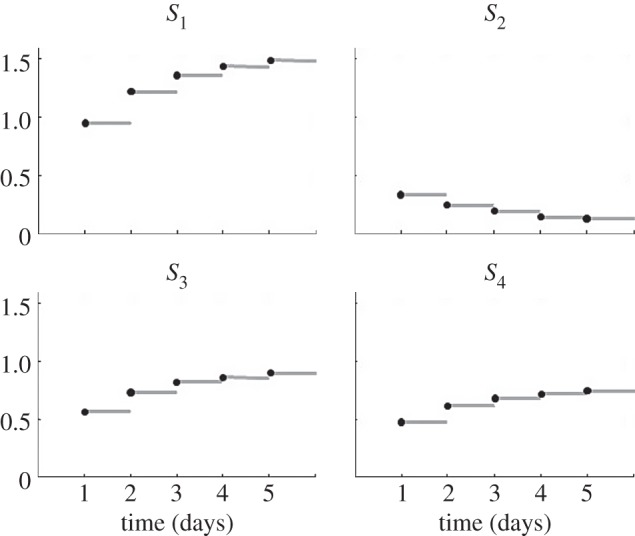
The steady states of the five models obtained in case 2 were simulated with the mean value of the ensemble of parameter *k*_1_ for the different stages.

In case 2, the observations and the five resulting models are treated independently. To illustrate the difference with ADAPT (case 3), the model was simulated with the mean value of the ensemble of parameter *k*_1_ for the different stages (figure 6). Since the model is parametrized for steady-state data at the different stages (the black dots), and further information on the dynamic behaviour in between these observations is lacking, five separate steady-state simulations have been performed.

### 5.3. Analysis of dynamic adaptations in parameter trajectories

**Figure 7.**
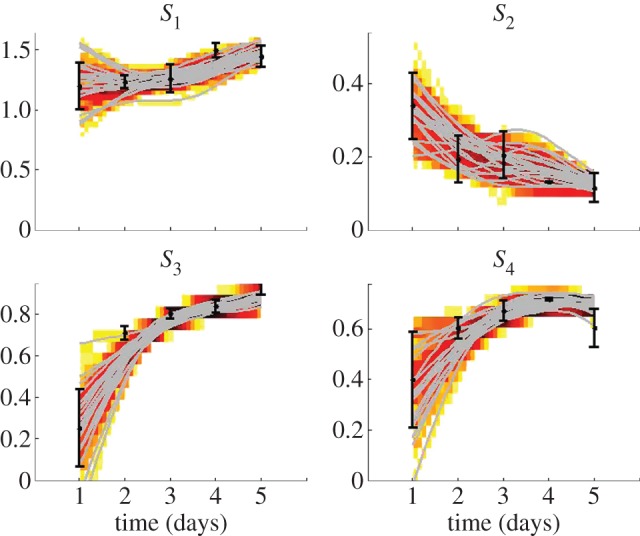
Data interpolants, sampled by bootstrapping of the data according to a Gaussian distribution with a mean and standard deviation as indicated with the dots and error bars, respectively. A subset of 20 of the interpolants is shown (grey lines). The complete distributions are shown as two-dimensional histograms. A darker colour represents a higher density of the splines in that specific region and time point. (Online version in colour.)

Figure 7 shows a subset of the spline interpolants fitted through the data. The splines provide a description of the data such that *X_d_* in equation (3.4) can be calculated for each time interval *δt* of the step-wise simulation with the time-varying parameters. The complete distributions are shown as two-dimensional histograms. A darker colour represents a higher density of the splines in that specific region and time point.

**Figure 8.**
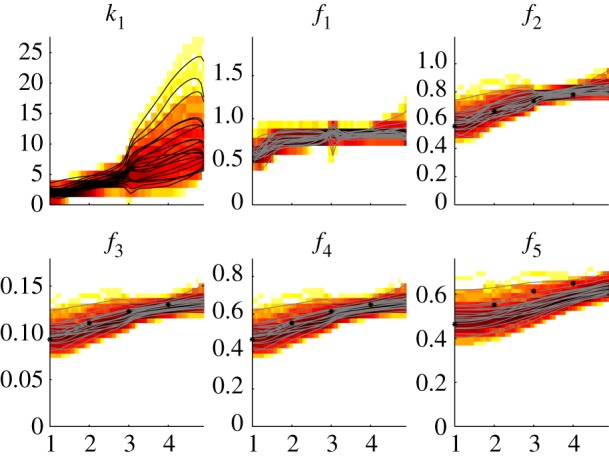
Case 3, results of ADAPT. A subset of the identified time-dependent descriptions of *k*_1_ (black) and the corresponding fluxes (grey) are shown. The complete distributions are shown as two-dimensional histograms. A darker colour represents a higher density of the trajectories in that specific region and time point. The values of the true fluxes, which are known in this theoretical study, have been included for comparison (*). (Online version in colour.)

The ADAPT results, including the parameter trajectory for *k*_1_(*t*) and corresponding five fluxes in the metabolic network, are shown in figure 8. The ADAPT results were consistent with the observations in cases 1 and 2. The size of fluctuations in the parameter trajectory is controlled by setting (tuning) *λ_r_*.

These results show that (i) the approach can infer accurate information about the molecular adaptations in the metabolic network, without having explicit information available about changes in gene expression and protein activity, (ii) it provides a model that can link different metabolic phenotypes, and (iii) provides an analysis of the uncertainty in model parameters and model results (the accuracy of the data description as well as predictions of unobserved quantities).

### 5.4. Application: variations in lipoprotein metabolism

**Figure 9.**
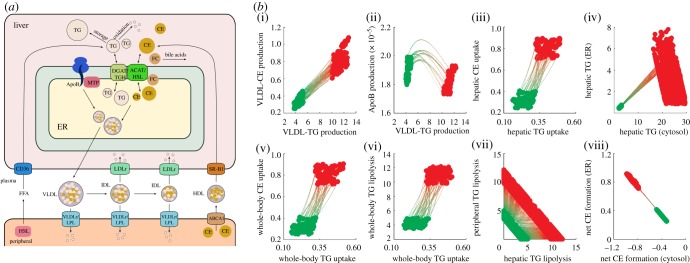
(*a*) Schematic overview of the model of hepatic lipid and plasma lipoprotein metabolism in mice. (*b*) Identified flux adaptations upon T0901317-induced LXR activation. Flux trajectories from the control phenotype (light grey) to the LXR-activated phenotype (dark grey). Molecular fluxes ((i)–(iii), (v)–(viii)) are given in mM/h, whereas the triglyceride concentrations presented in (iv) are given in mM (for details, see [10]). Figures adapted from Tiemann *et al.* [10]. (Online version in colour.)

To show the relevance of the ADAPT approach for real-life applications, we discuss its recent application in a biomedical study investigating changes in hepatic lipid and plasma lipoprotein metabolism [10]. Understanding the metabolism of apoB containing very low-density lipoprotein (VLDL) and apoA containing high-density lipoprotein (HDL) particles is of high importance, as these are risk factors for metabolic syndrome and associated cardiovascular diseases [26,27]. In this study, changes in lipoprotein metabolism were induced by activating liver X receptor (LXR) in mice during a period of several days [12]. The LXR family (LXRα and LXRβ) are nuclear receptors and plays a central role in the control of cellular lipid and sterol metabolism. We were able to quantitatively integrate data of control mice as well as mice with activated LXR into a computational model (figure 9*a*). The model has three compartments representing the liver, blood plasma and peripheral tissues. The liver compartment includes reactions representing the production, utilization and storage of triglycerides and cholesterols, and the mobilization of these metabolites to the endoplasmic reticulum (ER), where they are incorporated into nascent produced VLDL particles. The VLDL particles are secreted in the plasma compartment where they serve as nutrients for peripheral tissues. Remnant particles are taken up and cleared by the liver. The model also includes the hepatic uptake of free fatty acids and reverse cholesterol transport via HDL. The model consists of eight ODEs and 24 fluxes. All metabolic reactions are described with mass actions kinetics and the model contains 24 parameters. Activation of LXR is known to affect most processes included in the model. However, many details on how LXR regulates these metabolic processes are unknown or unclear. With the current knowledge, it is impossible to mechanistically describe how changes in LXR result in changes in transcription and translation of the target genes and protein expression (including many post-translational modifications). ADAPT was successfully applied to infer and analyse the changes in the metabolic network during a 4-day intervention in which LXR was activated by an agonist.

As can be seen in figure 9*b*, several trajectories were very constrained, whereas others showed a large variability. Uncertainty in the model is associated with a wide distribution of acceptable values for some parameters and in some cases with multimodality. These distributions are different for the wild-type and LXR-activated phenotypes. With a modelling and parametrization approach such as applied in case 2 in the theoretical study differences in the distributions of the parameter estimates for the two datasets can be analysed. With ADAPT, it can also be traced how these parameter distributions are related and dynamically adapt during the phenotype transition. Moreover, there were also situations in which the data of the LXR-activated phenotype could not be successfully described by direct fitting of the data (i.e. without using ADAPT, appendix B). In appendix B, more information can be found on how the parameters of the ADAPT algorithm were chosen.

This study also showed that in addition to analysing progressive changes in metabolic concentrations and fluxes, the ADAPT approach can be useful for pharmaceutical research to study and identify the mode of action of (new) pharmaceutical compounds at the system level. To activate LXR, the mice were treated with LXR agonist T0901317, which has been considered as an interesting compound for new medication to halt the development of atherosclerosis. Using ADAPT, it was understood how on one hand LXR activation increases cholesterol efflux (whole-body uptake of cholesteryl ester, CE, figure 9*b*(v)), the anti-atherosclerotic mode of action, but simultaneously results in accumulation of fatty acids in the liver (hepatic triglycerides, TG, figure 9*b*(iv)). Hepatic steatosis is a serious side-effect which has hampered clinical testing of the compound. Hence, a potential application of the ADAPT approach is the prediction and analysis of the effectiveness of pharmacological interventions to treat progressive diseases.

## 6. Discussion

We presented applications of a recently developed, generic computational workflow employing mathematical modelling to predict the long-term metabolic adaptations in the development and progression of multi-factorial diseases, or after a therapeutic intervention. In these conditions, the metabolic parameters and fluxes can generally not be considered constant in time. ADAPT identifies which metabolic parameters and consequently fluxes necessarily have to change to describe the dynamics of the experimental data collected at different stages of a disease or treatment intervention. Here the ADAPT approach was tested in a theoretical study. Our approach was shown to be particularly useful to study biological systems from which the network topology is relatively well known, e.g. for metabolic pathways. The modulating effects on these pathways via interactions with the proteome and transcriptome, which are less well understood, could be captured by the time-dependent descriptions of the parameters. The concept of time-dependent model parameters was discussed as a way to describe the dynamics of molecular adaptations consistent with the available metabolic data. The case study concerned a relatively simple example, but therefore the results could be easily analysed. Its main purpose was to explain the concept behind ADAPT and provide insight in the approach. The relevance of ADAPT for real-life problems in which a disease or intervention affects many metabolic processes simultaneously was demonstrated by the analysis of progressive changes in lipoprotein metabolism induced upon pharmacological treatment of mice by LXR agonist T0901317.

### 6.1. Comparison and relation with other modelling approaches

ADAPT is based on ODEs, a common modelling approach in systems biology. Many of the well-established methods for simulation, analysis and parametrization of ODE models can be used with ADAPT. Modelling in molecular systems biology typically focuses on molecular pathways and mechanisms at the sub-cellular level (‘bottom-up’). These models are constructed to describe processes at a single time scale, usually capturing short-term dynamics ranging from seconds to hours [4,6,7]. This is, in general, also the case for ‘top-down’ approaches for modelling of biological systems. For example, compartment modelling to describe whole-body glucose homeostasis in diabetes research [7,28], and pharmacokinetic–pharmacodynamic modelling of the distribution, metabolism and biological effects of drugs used in pharmacology. In these models, the physiology is described in an empirical fashion, and body compartments and biological processes are treated with highly lumped approximations. To simulate the model and make predictions, parameter values are typically estimated from experimental data. In these kinetic models, inferred parameters and model results are constrained by the network topology, kinetic equations of the molecular processes and experimental data. Another important class of models applied in systems biology are constrained-based modelling approaches, e.g. genome-scale metabolic models (GSMM). By applying methods such as flux balance analysis (FBA), these models describe steady-state flux distributions under different physiological conditions or in different tissues [2]. Constrained-based modelling primarily exploits the constraints imposed by the structure of the metabolic network, without information about the kinetics and dynamics.

An extension of FBA with attempts to describe dynamic changes in metabolic network is dynamic flux balance analysis (dFBA, [29,30]). dFBA describes time-varying fluxes, but does not include kinetic equations. Another approach in which constant quantities in a simpler modelling approach are made time-varying to describe more complicated systems is linear parameter-varying (LPV) models. LPV is a class of systems studied in systems and control theory [31,32], and primarily used to design nonlinear controllers. To our knowledge, LPV models have not been applied in modelling of biomolecular networks.

The dynamic trajectories of model parameters as well as concentrations and fluxes obtained by ADAPT are constrained by the network topology, kinetic equations of the molecular processes and data. As such, our method exploits and integrates the merits from constrained-based modelling approaches to describe steady-state conditions and kinetic modelling for system dynamics. Similar to GSMM, ADAPT focuses on a level of biological systems of which we, in general, have particular good insights, metabolism, and combines aspects from different approaches. Hence, it could be referred to as a ‘middle-out’ approach.

### 6.2. Advantages of ADAPT

Several of the merits of ADAPT are discussed here.

— In case of multi-factorial, progressive diseases, but also for fundamental biological processes, such as development and adaptation, the dynamic interaction between different biological levels (transcriptome, proteome, metabolome) needs to be considered and understood. With ADAPT, adaptations in the metabolic network can be described without the necessity to develop detailed kinetic models of the modulating mechanisms (e.g. changes in gene expression, modified protein activity through changes in phosphorylation). The parameter trajectories provide coarse-grained approximations of these levels of regulation. However, with currently available information on gene and protein interaction networks attempts to include a mechanistic description of these interactions and how they regulate metabolism will also result in models that can only be crude approximations.
— ADAPT provides an approach to develop and analyse models of processes involving time-scales that are orders of magnitude different. In preclinical and clinical research, data are typically collected after an overnight fast. The aim is to study the metabolic system in a (pseudo) steady-state condition that is reproducible on a day-to-day basis. The models can describe short-term dynamics (in the order of seconds) of a metabolic network in response to a metabolic variation, for example, a small, rapid change in blood plasma glucose. The same model can also describe the long-term adaptations induced by a progressing disease or intervention. An important benefit of ADAPT is that it enables analysis of changes in metabolic control, including the prediction of the short-term dynamics, at any time during the progressive adaptation. This includes stages in between the experimentally observed phenotypes.
— Because of the uncertainties associated with the data and the complexity of the system at hand, in many systems biology models multiple parameter sets can be inferred that adequately describe the data. In case of ADAPT, in general multiple acceptable parameter transition trajectories are obtained. Analysing how uncertainties in experimental data propagate through parameter values and model predictions is an increasingly important topic in systems biology [8,9]. Undermodelling can be an important source of errors in the model and results in a bias in parameters values. An important merit of the ADAPT framework is that it accommodates different types of uncertainties associated with modelling of biological systems. In the design and development of the approach, both the uncertainty arising from natural, unpredictable variation in the (observations of the) system under study, as well as uncertainty due to the lack of knowledge about the system have been considered.
  To account for uncertainties a bootstrapping approach was used. Replicates of the observed data were sampled from a parametric error model distribution, and the estimation was repeated for each of these samples. Moreover, the optimization procedures were repeated for a dispersed range of initial parameter values (Monte Carlo multiple minimization), which enables to probe the parameter space for the existence of multiple minima [8]. Alternatively, a Bayesian approach could have been used to infer the probability density of parameter values and model results [8,9]. The resulting variations in parameter trajectories, fluxes and concentrations are also dependent on the regularization included in the objective function. Via a Pareto analysis (appendix B) a trade-off can be made between accuracy of the data fit versus some *a priori* knowledge, assumption or hypothesis. Here, it was assumed that progressive adaptations in metabolic networks correspond to smooth parameter trajectories. However, this is not a requirement of our approach. Other prior knowledge can be incorporated through regularization as well. For example, if gene expression data are available, one could select for parameter trajectories that correlate with changes in gene expression.
— Another advantage of ADAPT is that it is not necessary to have the same type of data (the same measurements) available for all time points (phenotypes). Missing data are a common situation in many datasets. This might make parametrization impossible in case of independent parameter estimation for each phenotype. Because of the data interpolation, it does not cause a fundamental problem for ADAPT.
— Independent parametrization for each phenotype (such as case 2 with the toy model) has to assume a steady state (figure 6). In case the true system is continuously changing, independent parametrization can result in inaccurate parameter estimates. ADAPT does not rely on a steady-state assumption.
— The computational implementation of ADAPT generates a collection of possible solutions reflecting the uncertainty in experimental data and the uncertainty in the model. This requires many iterative simulations of the model. To effectively calculate the solutions software optimized for high-performance computing was used. Once the ensembles of trajectories of metabolic states, parameters and fluxes have been obtained it opens the possibility for a wide range of analysis techniques [13]. For example, analyses which require a time-course, such as the calculation of a total production (integral of certain fluxes), or the rise and fall periods of certain adaptations. Analyses of the differences in the trajectories might identify biologically different scenarios of disease progression, critical transition thresholds and possible biomarkers for improved patient stratification. Ultimately such models could contribute to the development of novel and/or patient-specific interventions.

### 6.3. Outlook

The development of ADAPT was motivated by the need to understand multi-factorial progressive diseases and to integrate and interpret multivariate phenotype data in clinical and preclinical research. Other applications are foreseen. The approach can also be applied in case the dynamics of the metabolic network and the time-scale of the unknown interactions with the transcriptome–proteome levels are of the same order of magnitude. For example, the glycolytic enzymes phosphofructokinase (PFK) and pyruvate kinase (PK) are regulated by allosteric effectors and by covalent modification. Phosphorylation of PFK and PK is regulated by the blood glucose level, mediated by glucagon and insulin. As many details are unknown, this regulation has not been incorporated in existing models of glycolysis [33–35]. ADAPT could offer a possibility to improve and extend these models.

In case of progressive metabolic diseases, such as type 2 diabetes and metabolic syndrome, many details of the underlying metabolic networks are known. The second source of information can be obtained from experimental data on metabolite concentrations and fluxes (metabolic profiling). Metabolic profiling of blood plasma, urine, but also tissues becomes increasingly feasible, using technological advances such as in (*in vivo*) nuclear magnetic resonance spectroscopy [36,37]. Metabolomics is expected to contribute significantly to the development of personalized healthcare and personalized medicine (also referred to as precision medicine, [38]). The metabolite profile of a subject constitutes the interaction of the genotype with the environment and therefore is the molecular reflection of the (clinical) phenotype. Hence, metabolomics has relevance for other diseases than only the ‘typical’ metabolic pathologies [39]. ADAPT could be one of the novel computational approaches necessary to structure and interpret the multivariate phenotype data and translate it in useful information and understanding.

## Figures and Tables

**Figure 10.**
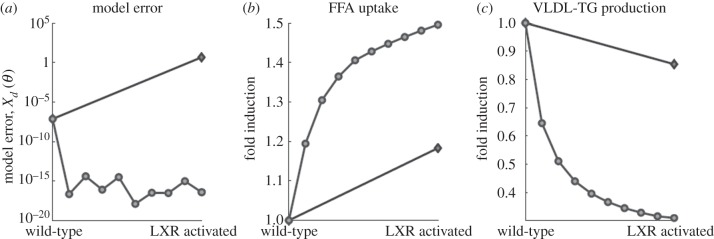
Effect of using a step-wise optimization. An example of an acceptable parameter set describing the wild-type phenotype, which was not successfully optimized by single-step optimization to describe the LXR-activated phenotype (diamonds). When ADAPT was applied the phenotype transition could be successfully described (circles). (*a*) The model error *X_d_*. (*b*,*c*) Two examples of corresponding parameter trajectories from the wild-type phenotype to the LXR-activated phenotype, normalized by the values of the parameters of the wild-type phenotype. Adapted from Tiemann *et al.* [10].

**Figure 11.**
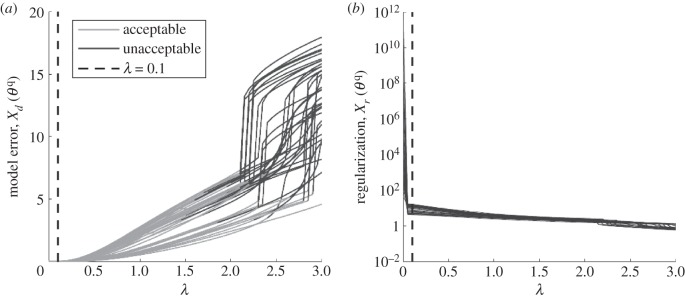
Effect of regularization strength *λ*. Here *λ* = √*λ_r_* is shown on the horizontal axis. (*a*) Model error *X_d_* for all acceptable parameter sets for increasing *λ*, where light grey indicates an acceptable data fit and dark grey an unacceptable data fit. (*b*) Regularization error *X_r_* for increasing *λ*, resulting in a so-called Pareto frontier. Any point on the frontier curve is Pareto efficient and it is up to the user to make trade-offs and select which *λ* is used. The dashed line indicates the *λ* that was selected (*λ_r_* = 0.01). Adapted from Tiemann *et al.* [10].

## Appendix A. Parameters, input fluxes and initial conditions

Tables 2–4.

**Table 2.** Parameters.

parameter	value
*V*_max_	1
*K_i_*	0.1
*k*_2_	1
*k*_3_	0.1
*k*_4_	0.5
*k*_5_	1
*k*_6_	0.01

**Table 3.** Input fluxes.

flux	value
*u*_1_	1
*u*_2_	1

**Table 4.** Initial conditions.

state variable	value
*S*_1_	1.03
*S*_2_	0.38
*S*_3_	0.62
*S*_4_	0.52
*R*_1_	0.52

## Appendix B. Effect of settings of ADAPT algorithm

ADAPT contains several algorithmic parameters (table 1). How these values should be chosen, is problem dependent, and their tuning involves some trial an error. Two of the settings are briefly discussed here for the model describing progressive changes and phenotype transitions in hepatic lipid and plasma lipoprotein metabolism.

**Table 5.** External perturbation to generate the mock data. The information in table 5 was not used in the algorithm (was considered unknown).

phase	parameter *k_d_*
1	10 × 10^−3^
2	4.2 × 10^−3^
3	1.8 × 10^−3^
4	0.7 × 10^−3^
5	0.3 × 10^−3^

The step-wise optimization used in ADAPT guides the parameter estimation algorithm to infer parameter trajectories that describe the phenotype transition. Hereby convergence of the minimization algorithm to local unacceptable minima can be prevented. Figure 10 shows an example of an acceptable parameter set describing the wild-type phenotype, which was not successfully reoptimized if the model was fitted directly to the data of the LXR-activated phenotype (i.e. the same approach as used in case 2 of the case study). This problem was circumvented by the multi-step optimization used in ADAPT.

The parameter trajectories were regularized according to equation (3.3) to avoid needless change of parameters. A trade-off has to be made between the two objectives in the optimization. For low *λ_r_*, the regularization term has no effect, whereas for large *λ_r_* the parameter estimation algorithm might focus on minimizing the regularization term while describing the experimental data inaccurately. Therefore, the effect of *λ_r_* on the sum of squared model errors *X_d_* and the sum of squared parameter differences *X_r_* was investigated for a collection of acceptable parameter sets for the transition from wild-type to LXR-activated phenotype. Figure 11*a* shows *X_d_* for increasing *λ_r_*, where light grey indicates an acceptable data fit and dark grey an unacceptable data fit. Figure 11*b* shows *X_r_* for increasing *λ_r_*. Note that a small *λ_r_* is already sufficient to minimize parameter changes, while the experimental data are still described very well. It is preferred to bias the data fitting as little as possible, and therefore a *λ_r_* of 0.01 was selected.
